# Unsupervised machine learning revealed a correlation between low-dose statins and favorable outcomes in ICH patients

**DOI:** 10.3389/fneur.2025.1573036

**Published:** 2025-06-24

**Authors:** Chaohua Cui, Haoye Guan, Tonghua Long, Yuru Liang

**Affiliations:** Life Science and Clinical Medicine Research Center, Affiliated Hospital of Youjiang Medical University for Nationalities, Baise, Guangxi, China

**Keywords:** unsupervised machine learning, intracerebral hemorrhage, low dose statin, mortality, cerebrovascular events

## Abstract

**Background:**

Statins were effective in preventing ischemic stroke in patients with intracerebral hemorrhage (ICH). Statins administered at normal doses may contribute to hematoma expansion. We employed unsupervised machine learning methods and found that low-dose statins improve the prognosis of ICH.

**Methods:**

We employed unsupervised machine learning techniques to analyze unidentified factors within a retrospective cohort related to the prognosis of ICH. In our prospective cohort study, data were gathered from ICH patients over a three-year period. Outcomes included mortality, cerebrovascular events within 3 years, unfavorable functional outcomes at 3 months, and hematoma expansion during hospitalization.

**Results:**

The heatmap illustrated distinct statin effect trajectories, emphasizing the substantial impact of low-dose statins on prognosis. The low-dose statin group exhibited reduced mortality (*p* = 0.001), a decreased rate of unfavorable functional outcomes (*p* < 0.001), and fewer cerebrovascular events (*p* < 0.001). Both groups demonstrated a comparable rate of hematoma expansion (*p* = 0.735). In multivariable Cox regression analyses, low-dose statins were associated with reduced mortality (RR = 0.505, *p* = 0.022) and decreased ischemic stroke occurrence (RR = 0.354, *p* < 0.001). Additionally, low-dose statins were linked to better functional outcomes (OR = 0.594, *p* < 0.001) but showed no significant association with hematoma expansion (OR = 1.056, *p* = 0.735).

**Conclusion:**

Unsupervised machine learning revealed a correlation between low-dose statins and patient prognosis. Subsequent analyses and validation indicated that for patients with intracerebral hemorrhage (ICH) in the cerebral hemisphere, early administration of low-dose hydrophilic statins is associated with reduced mortality, and statins are associated with a reduced rate of ischemic stroke occurrence. For all ICH patients, low-dose statins are associated with a lower rate of unfavorable functional outcomes but show no significant association with hematoma expansion.

## Introduction

Ischemic stroke is a common complication in patients with intracerebral hemorrhage (ICH) (1). Ischemic stroke can exacerbate the condition of ICH patients and increase their mortality rate (1). Antiplatelet drugs and statins are guideline-recommended interventions for preventing ischemic stroke (2). However, antiplatelet medications are contraindicated for patients with ICH. Consequently, statins remain the sole pharmacological intervention available for ischemic stroke prevention in ICH patients. ICH patients may benefit from the neuroprotective mechanisms of statins (2).

Statins may increase the incidence of ICH according to a large cohort study (3). One study indicated that statin use in hospitals may contribute to hematoma expansion in ICH patients (4). Another study indicated that higher LDL-C levels are associated with lower rates of hematoma expansion and mortality (5). These findings raised concerns regarding the safety of statins for ICH patients. One study found no association between statins and hematoma expansion (6). Several studies have demonstrated that statins can reduce mortality in ICH patients (7, 8). Our previous study found that low-dose statins can prevent ischemic stroke without increasing the incidence of ICH in ischemic stroke patients (9).

Our previous retrospective cohort analysis revealed contradictory findings; while ICH patients using statins appeared to have better outcomes, these conclusions were unstable. Consequently, we utilized unsupervised machine learning techniques to identify additional factors influencing patient outcomes and discovered that varying statin doses further impacted patient prognosis; those receiving low-dose statins exhibited improved outcomes. Furthermore, one study demonstrated that low-dose statins decrease mortality in ICH patients (8). However, the previous study included only 98 patients and was retrospective, lacking clinical robustness. Thus, a prospective study was warranted to include a larger cohort and more comprehensive outcomes to thoroughly investigate the relationship between low-dose statins and ICH patients.

We conducted a prospective study involving a larger cohort of patients receiving low-dose statins. Our study investigates the relationship between low-dose statins and mortality, cerebrovascular events, hematoma expansion, and functional outcomes in ICH patients. Additionally, we examine whether varying statin characteristics and clinical factors influence this relationship.

## Methods

### Cohort 1: retrospective explored cohort

#### Patients

The study cohort was retrospectively observational in nature. It comprised consecutive patients diagnosed with ICH. Patients were recruited from the neurosurgery, neurology, and rehabilitation departments of the Affiliated Hospital of Youjiang Medical University for Nationalities between June 1, 2018, and May 30, 2019.

The inclusion criteria were as follows: (1) patients aged 18 years and above, (2) a mandatory computed tomography (CT) scan for index ICH admission within 24 h of symptom onset, and (3) receipt of conventional medical treatment and rehabilitation therapy following admission. The exclusion criteria included: (1) presence of malignant tumors, (2) diagnoses of secondary ICH, encompassing intracerebral aneurysm, cerebral arteriovenous malformation, Moyamoya disease, brain tumor, and hemorrhagic transformation due to brain infarction, and (3) a pre-existing modified Rankin Scale (mRS) score exceeding 2.

#### Data collected

Demographic characteristics (age, gender), treatment methods (antihypertensive, statins, etc.), duration of bed rest, and laboratory data were extracted from electronic clinical records. Medical history, including stroke, hypertension, and smoking status, was obtained through structured questionnaires completed by patients or their relatives. Patients’ NIHSS (National Institutes of Health Stroke Scale) and mRS (Modified Rankin Scale) scores were recorded upon admission and at discharge. Patients’ GCS (Glasgow Coma Scale) and ADL (Activities of Daily Living) scores were documented at admission.

#### Classify data by unsupervised machine learning

Python 3.8 was employed for the analysis. During the methodological exploration for risk factor analysis, three unsupervised machine learning approaches—K-means clustering, hierarchical clustering, and Gaussian Mixture Models—were rigorously tested prior to selecting the final method. Upon determining that K-means clustering and Gaussian Mixture Models were insufficient for effective dataset evaluation, hierarchical clustering was identified as the most robust analytical approach owing to its capability to manage complex variable interrelations via dendrogram visualization and customizable similarity metrics.

Initially, for cluster analysis, all data were standardized utilizing the StandardScaler module from the sklearn library, followed by classification using Hierarchical Clustering methods, specifically the AgglomerativeClustering module from the sklearn library. The heatmap depicted distinct characteristics within the classified patient groups. Outcome events were compared across the distinct identified groups. Grouping can provide clinical significance by elucidating differences in outcomes among patient groups. Subsequently, chi-square tests and *t*-tests were employed to further analyze factors within the selected groups. Significant differences were observed in low-dose statin use across the various groups. Subsequently, the impact of low-dose statin use on the outcomes of ICH was validated.

### Cohort 2: prospective validated cohort

#### Study subjects

The study cohort is a prospective observational cohort. ICH patients were recruited from the neurosurgery, neurology, and rehabilitation departments of two hospitals affiliated with Youjiang Medical University for Nationalities and Baidong Hospital of Youjiang Medical University for Nationalities. Enrollment of patients occurred between January 1, 2020, and October 1, 2021, with follow-up extending until October 1, 2024.

Patients administered low-dose statins post-admission were categorized into the statins group, while remaining patients were categorized into the no-statin group. Low-dose statins were defined as daily administration of atorvastatin 10 mg, simvastatin 10 mg, pitavastatin 1 mg, lovastatin 20 mg, and rosuvastatin 5 mg following onset. Based on previous research, the study requires recruitment of over 200 patients utilizing low-dose statins to ensure adequate statistical power.

Inclusion criteria encompassed ICH patients aged 18 years or older who underwent head CT examinations and met WHO ICH diagnostic standards. Exclusion criteria included traumatic cerebral hemorrhage, ICH due to venous sinus thrombosis, metastatic lesions, or underlying vascular abnormalities. The study excluded patients taking other statin dosages or alternative lipid-lowering therapies due to their limited number (*n* = 13).

The study was performed in accordance with the Declaration of Helsinki and the ethical standards of the institutional and/or national research committee. The Ethics Committee of the Affiliated Hospital of Youjiang Medical University for Nationalities approved the study. Informed consent was obtained from all participants or their surrogates in the study.

#### Clinical data

Risk factors were included based on established guidelines, supplemented by clinically significant factors. Patient demographic, vital sign, and laboratory data were extracted from electronic clinical records. Demographic data encompassed age, gender, nationality, occupation, and other variables (Table 1). Vital signs comprised heart rate, blood pressure, and body temperature upon admission. Laboratory data included PLT (platelet count), INR (international normalized ratio), hemoglobin, TC (total cholesterol), TG (triglycerides), HDL-C (high-density lipoprotein cholesterol), LDL-C, and other parameters (Table 1). Patients’ medical and medication histories were collected using structured questionnaires completed by either the patients or their relatives. NIHSS (National Institute of Health Stroke Scale), mRS (Modified Rankin Scale), GCS (Glasgow Coma Scale), and ADL (Activity of Daily Living) scores were assessed upon patient admission.

**Table 1:** Baseline characteristic and outcome data by univariate analysis.

Risk factor	Low dose statin group (*N* = 669)	No-statin group (*N* = 479)	P*
Baseline characteristic			
Age, years	62.59 (13.437)	63.65 (13.430)	0.185
Female, %	248 (37.1)	177 (37.0)	0.967
Admission NIHSS score	9 (3–16)	10 (3–18)	0.293
Admission mRS score	4 (2–4)	4 (2–4)	0.460
Admission GCS score	13.50 (2.680)	13.25 (2.262)	0.095
Admission ADL score	40 (20–80)	40 (25–70)	0.319
Admission hematoma, ml	20 (11–23)	20 (10–25)	0.345
SBP at admission, mmHg	147.93 (26.334)	148.47 (27.749)	0.738
DBP at admission, mmHg	86.59 (16.227)	83.90 (16.947)	0.759
Heart rate at admission	82.50 (12.436)	83.25 (14.980)	0.378
Body temperature at admission	36.64 (0.469)	36.67 (0.497)	0.210
Taking antithrombotic agents	243 (36.3)	157 (32.8)	0.201
History of statins	103 (15.4)	82 (17.1)	0.143
History of hypertension, %	410 (61.3)	313 (65.3)	0.160
History of diabetes Mellitus, %	142 (21.2)	107 (22.3)	0.652
History of atrial fibrillation	113 (16.9)	88 (18.4)	0.435
History of ischemic stroke, %	68 (10.2)	48 (10.0)	0.845
History of ICH, %	78 (11.7)	72 (15.0)	0.122
Carotid atherosclerosis, %	237 (35.4)	111 (23.2)	**<0.001**
pneumonia, %	223 (33.3)	186 (38.8)	0.055
epilepsy, %	27 (4.0)	23 (4.8)	0.531
Antiinfection drug, %	240 (35.9)	199 (41.5)	0.051
Left cerebral hemisphere, %	345 (51.6)	221 (46.1)	0.085
Platelet, mmol/l	202.03 (66.694)	204.79 (76.694)	0.516
INR	1.04 (0.147)	1.06 (0.229)	0.071
Hemoglobin, mmol/l	128.34 (19.671)	127.53 (21.754)	0.416
Albumin, mmol/l	36.52 (6.358)	36.84 (6.759)	0.404
ALT, mmol/l	25.97 (19.847)	23.73 (19.685)	0.259
AST, mmol/l	26.17 (15.072)	27.22 (17.589)	0.279
Creatinine, mmol/l	81.71 (43.646)	84.13 (77.092)	0.502
Glucose, mmol/l	7.61 (3.118)	7.73 (3.249)	0.526
Triglyceride, mmol/l	1.58 (1.201)	1.47 (0.919)	0.089
Total cholesterol, mmol/l	4.19 (1.097)	4.24 (0.998)	0.434
HDL-C, mmol/l	1.26 (0.372)	1.24 (0.368)	0.357
LDL-C, mmol/l	2.42 (0.913)	2.49 (0.805)	0.163
Outcome data			
Mortality, %	143 (21.4)	145 (30.3)	**0.001**
cerebrovascular events, %	87 (13.0)	102 (21.3)	**<0.001**
UFO, %	283 (42.3)	271 (56.6)	**<0.001**
Hematoma expansion, %	114 (17.0)	78 (16.3)	0.735

P* was calculated by ANOVA, Chi-square test, or Mann–Whitney U test as appropriate. NIHSS, National Institute of Health stroke scale; mRS, Modified Rankin Scale; GCS, Glasgow Coma Scale; ADL, activity of daily living; SBP, Systolic Blood Pressure; DBP, Diastolic Blood Pressure; ICH, intracerebral hemorrhage; INR, international normalized ratio; ALT, glutamic-pyruvic transaminase; AST, glutamic oxalacetic transaminase; HDL-C, high-density lipoprotein cholesterol; LDL-C, low-density lipoprotein; UFO, unfavorable function outcome. *P* values highlighted in bold signify statistically significant results.

### Statin use

We gathered data on statin usage among patients during hospitalization and follow-up. Statin types included atorvastatin, rosuvastatin, simvastatin, among others. Statin initiation was categorized into early use (within 7 days post-admission) and late use (beyond 7 days post-admission). Statin compliance was categorized into continuous use (adherence >80%) and discontinuous use (adherence <80%). Statins were classified into lipophilic (atorvastatin, simvastatin, pitavastatin, lovastatin) and hydrophilic (rosuvastatin) categories.

### Outcome events

The primary outcomes included mortality and cerebrovascular events occurring within a three-year period. Data on in-hospital mortality and cerebrovascular events were extracted from electronic clinical records. Post-discharge mortality and cerebrovascular event data were collected via telephone (using two distinct patient or relative numbers), WeChat (an instant messaging application), or email. Additionally, the types of cerebrovascular events (ischemic stroke or ICH) were documented. Outcomes were assessed by two neurologists who were blinded to patient condition and group allocation.

Secondary outcomes included unfavorable functional outcome (UFO) at 6 months and hematoma expansion during hospitalization. Unfavorable functional outcome was defined as mRS > 2 at 180 days post-admission. mRS scores were obtained through face-to-face interviews. Hematoma volume was assessed using Alice (PAREXEL International, Waltham, MA, United States). ICH volumes were determined using the ABC/2 method. Initially, hematoma volume was assessed via head CT at admission. Subsequently, hematoma volume was assessed from head CTs conducted during early hospitalization (3 to 7 days). Hematoma expansion was defined as an increase in volume of more than 33% compared to the initial measurement. Volume assessments were conducted by two neurologists blinded to patient condition and group allocation. The intraclass correlation coefficient for hematoma volume assessments between the two neurologists was 0.96.

### Statistical analysis

Statistical analyses were conducted using SPSS 23.0 for Windows, R 4.1.3, and Python 3.8.

#### Baseline characteristics

A *t*-test was employed for continuous variables (e.g., blood pressure, heart rate, laboratory measures) that were normally distributed between the two groups. These variables were expressed as mean ± standard deviation (SD). The Mann–Whitney U test was applied to continuous variables (e.g., NIHSS, ADL, GCS) that were non-normally distributed between the two groups. These variables were expressed as medians and interquartile ranges. The chi-square test was utilized for categorical variables (e.g., gender, disease history, medication history) and ordinal variables (e.g., mRS, occupation, nationality) between the two groups. These variables were presented as frequencies and percentages. Statistical significance was defined as *p* < 0.05.

#### Outcome variables

The chi-square test was utilized to compare mortality, cerebrovascular events, unfavorable functional outcomes, and hematoma expansion between the two groups. Primary outcomes included mortality and cerebrovascular events, both recorded over a three-year observation period. Given the binary nature of these endpoints and their time-to-event characteristics, the Cox proportional hazards model was deemed highly suitable for analyzing these composite outcomes in survival analysis. Cox regression methods were employed to analyze the relationship between risk factors, mortality, and cerebrovascular events across different time points. Logistic regression methods were applied to investigate the association between risk factors and unfavorable functional outcomes as well as hematoma expansion.

Eligible factors for multivariable Cox or logistic regression were screened as follows: (1) *p*-values from univariable Cox or logistic regression < 0.05; (2) LASSO regression was used to select eligible factors; (3) factors identified from steps (1) and (2) were included; (4) clinically significant factors were supplemented. Subsequently, multivariable Cox or logistic regression methods were applied to analyze eligible data. Rate ratios (RR), odds ratios (ORs), 95% confidence intervals (CIs), and *p*-values were calculated using Cox or logistic regression methods. Kaplan–Meier (K-M) curves were utilized to compare mortality and cerebrovascular events across different groups.

Subgroup analyses were performed based on various statin characteristics and key clinical features. Groups were subdivided based on different statin initiation times, statin compliance, statin type, and ICH location (cerebral hemisphere, including thalamus, vs. brainstem), as fewer patients were present in the thalamus and other locations. Additional subgroup analyses included stratifications based on carotid atherosclerosis and stroke type for cerebrovascular events. Subgroup results were presented using forest plots generated with the forestplot package in R.

## Results

### Cohort 1: retrospective explored cohort

**Figure 1:**
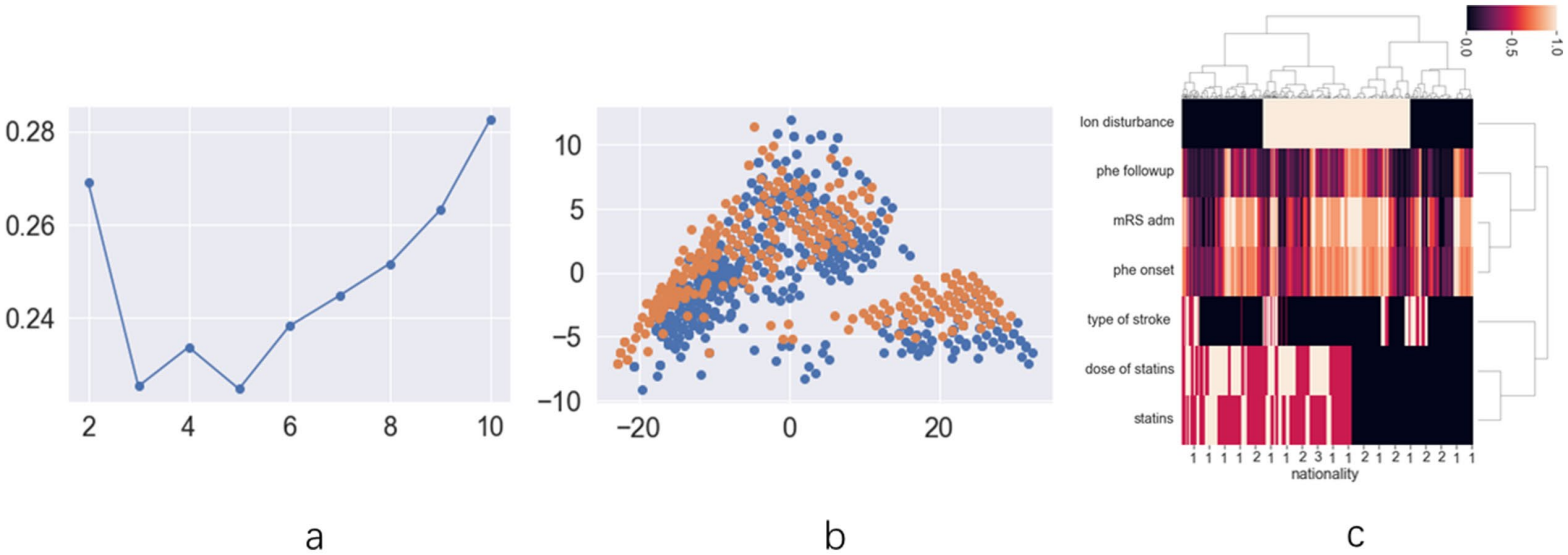
The results of unsupervised machine learning for all patients in retrospective cohort: **(a)** the line chart of silhouette score demonstrates that dividing the data into two clusters represents the best grouping choice; **(b)** The two-dimension scatter plot of suggests that the characteristic features among different groups lack clear differentiation; **(c)** The hot map for hierarchical clustering methods demonstrates limited differentiation in statin distribution patterns between distinct prognostic groups.

**Figure 2:**
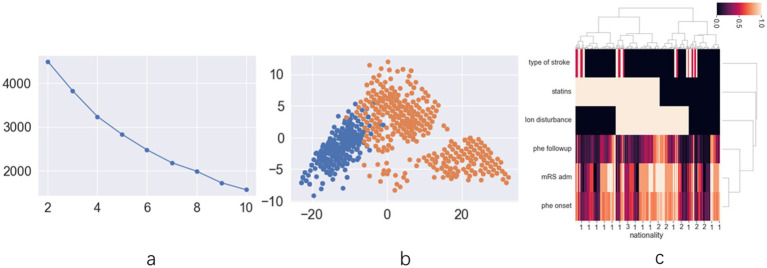
The results of unsupervised machine learning for low dose statins patients in retrospective cohort: **(a)** the line chart of silhouette score demonstrates that dividing the data into two clusters represents the best grouping choice; **(b)** The two-dimension scatter plot demonstrates clear and distinct differentiation of data characteristics across different groups; **(c)** The hot map for hierarchical clustering methods indicates distinct statin distribution across various prognosis groups.

The exploratory cohort comprised 191 patients (Supplementary Figure 1). Ninety-one patients were female (33.5%), with a mean age of 63.21 ± 13.74 years. Upon analyzing patient data across all statin dosages, score plots suggested that the optimal classification was either binary or quintuple (Figure 1a). The scatter plot for binary classification showed overlapping points, rendering the groups indistinct (Figure 1b). The heatmap similarly revealed indistinct statin effect lines, although dosage exhibited a clearer impact (Figure 1c). Focus was then shifted to patients using low-dose statins or none, where score plots clearly indicated binary classification as optimal (Figure 2a). In this context, the scatter plot demonstrated clear group separation (Figure 2b), and the heatmap highlighted distinct statin effect lines, indicating a significant low-dose impact (Figure 2c).

Consequently, patients were classified into two groups (HCgroup1 and HCgroup2) using hierarchical clustering methods. When comparing mortality rates between the two groups, HCgroup1 (21.4%) exhibited a significantly lower rate (*p* = 0.001) compared to HCgroup2 (30.3%). In comparing ICH recurrence rates, HCgroup1 (13.0%) demonstrated a significantly lower rate (*p* < 0.001) than HCgroup2 (21.3%). Therefore, hierarchical clustering proved to be a more effective choice for the exploratory cohort. Comparison of data between HCgroup1 and HCgroup2 revealed that a higher proportion of patients in HCgroup1 were taking low-dose statins (29.4% vs. 10.4%, *p* < 0.001) and had carotid atherosclerosis (35.4% vs. 23.2%, *p* < 0.001). Other characteristics, such as NIHSS score, GCS score, mRS score at admission, age, gender, and hematoma volume at admission, did not exhibit significant differences between the two groups. These findings indicate that low-dose statins significantly impact mortality and recurrence rates among ICH patients. However, the association between increased low-dose statin usage and reduced mortality and recurrence rates necessitates further validation in studies with larger patient populations and prospective cohorts.

### Cohort 2: prospective validated cohort

#### Study subjects

Initially, 1,233 eligible patients were recruited for the validated cohort. Of these, 59 patients were lost to follow-up or had missing data on statin treatment, and 26 withdrew from the study. Ultimately, 1,148 patients were included in the study (669 in the low-dose statin group and 479 in the no-statin group) (Supplementary Figure 1). The mean age of the 1,148 patients was 63.03 ± 13.44 years, with 37.0% being female. The median NIHSS score at admission was 9 (interquartile range: 0–39). Compared to the no-statin group, the low-dose statin group exhibited a higher prevalence of carotid atherosclerosis at admission. No significant differences in other baseline characteristics were observed between the two groups (Table 1).

#### Mortality and stroke recurrence analysis using chi-square test

The low-dose statin group exhibited a lower mortality rate (21.4%) compared to the no-statin group (30.3%) (*p* = 0.001). The low-dose statin group demonstrated a lower rate of ICH recurrence (9.6%) compared to the no-statin group (12.3%) (*p* < 0.001). Additionally, the low-dose statin group showed a lower rate of ischemic stroke occurrence (3.4%) compared to the no-statin group (9.0%) (*p* < 0.001).

#### Cox regression analysis for mortality

The multivariable Cox regression analysis for mortality included age, gender, low-dose statin usage, mRS at admission, hematoma volume at admission, pneumonia, among other factors (Tables 1, 2). The analysis revealed that advanced age (RR = 1.015, *p* = 0.003) and higher mRS scores (RR = 1.551, *p* < 0.001) were associated with increased mortality. Low-dose statin usage (RR = 0.496, *p* = 0.019) was inversely related to mortality (Table 2). Kaplan–Meier curves indicated that the low-dose statin group exhibited lower mortality compared to the no-statin group across various time points (Supplementary Figure 2).

**Table 2:** Multivariate COX regression for mortality.

Risk factor	RR (95%CI)	P*
Using low-dose statin	0.496 (0.276–0.892)	**0.019**
Older	1.015 (1.005–1.024)	**0.003**
Female	1.094 (0.852–1.406)	0.481
Higher mRS score at admission	1.551 (1.219–1.972)	**<0.001**
Higher ADL score at admission	0.992 (0.981–1.003)	0.136
More hematoma at admission	0.961 (0.910–1.015)	0.155
Pneumonia	2.635 (0.918–7.560)	0.072

P* was calculated by multivariate COX regression. mRS, Modified Rankin Scale; ADL, activity of daily living. *P* values highlighted in bold signify statistically significant results.

**Figure 3:**
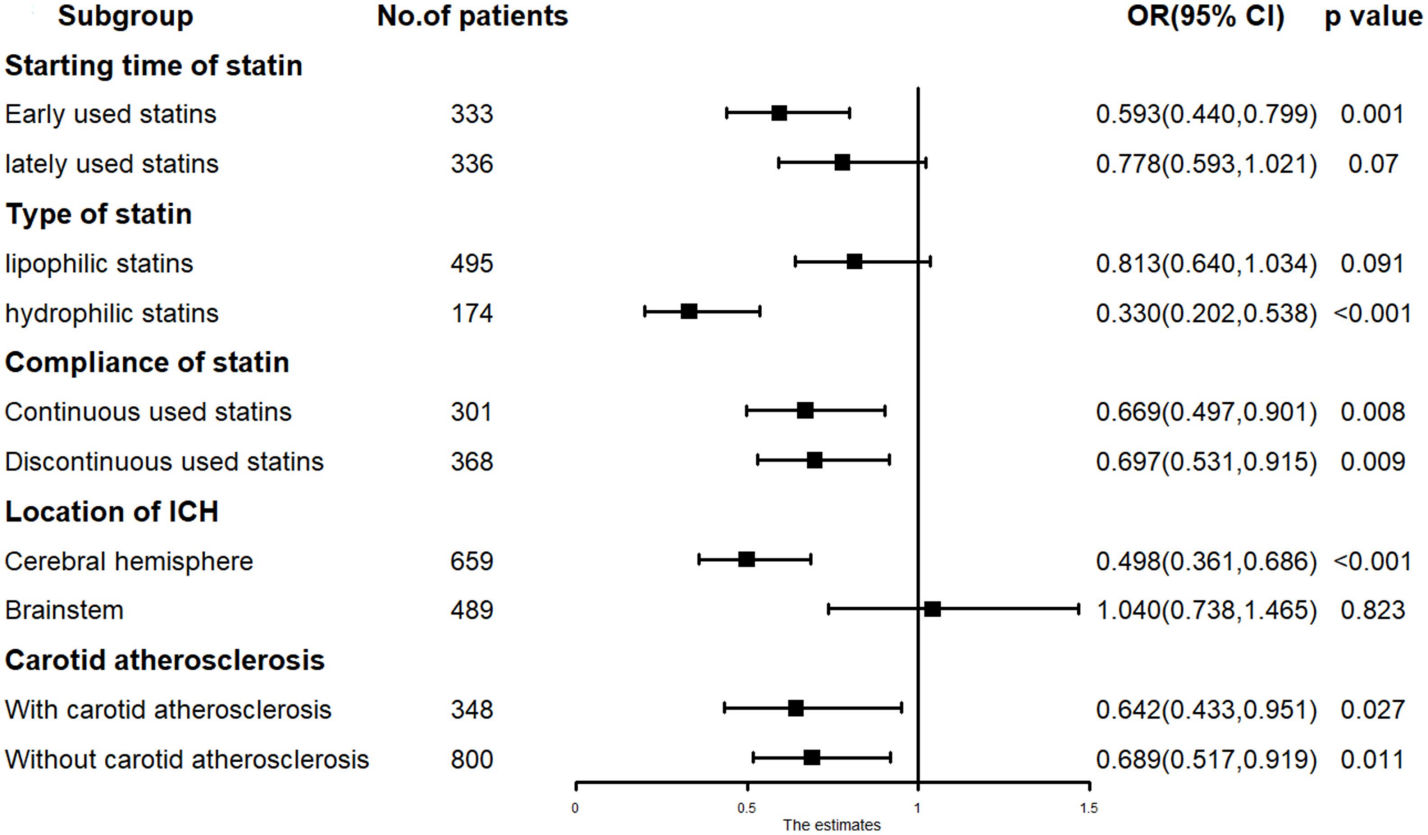
The subgroup analysis results in COX regression: low-dose statin related to mortality in hydrophilic statins, early-used and cerebral hemisphere subgroup.

Subgroup analysis revealed that early administration of statins (RR = 0.593, *p* = 0.001) was inversely related to mortality, whereas late administration (RR = 0.778, *p* = 0.070) showed no significant relation to mortality. Lipophilic statins (RR = 0.813, *p* = 0.091) were not significantly associated with mortality, whereas hydrophilic statins (RR = 0.330, *p* < 0.001) were inversely related to mortality. In cases of ICH located in the cerebral hemisphere, low-dose statin usage (RR = 0.498, *p* < 0.001) was inversely associated with mortality. In cases of ICH located in the cerebral brainstem, low-dose statin usage (RR = 1.040, *p* = 0.823) showed no relation to mortality. Significant relationships were observed in other subgroups (Figure 3).

#### Cox regression analysis for cerebrovascular events

The multivariable Cox regression analysis for cerebrovascular events included age, low-dose statin usage, NIHSS score at admission, GCS score at admission, platelet count (PLT) at admission, albumin levels at admission, carotid atherosclerosis, among other factors (Table 3). The results indicated that a higher NIHSS score (RR = 1.076, *p* < 0.001) at admission, advanced age (RR = 1.026, *p* = 0.001), carotid atherosclerosis (RR = 1.756, *p* < 0.001), and elevated albumin levels (RR = 1.029, *p* = 0.047) at admission were associated with an increased rate of cerebrovascular events. Conversely, low-dose statin usage (RR = 0.392, *p* < 0.001), a higher GCS score (RR = 0.899, *p* = 0.005) at admission, and elevated platelet count (RR = 0.996, *p* = 0.012) at admission were inversely associated with the rate of cerebrovascular events (Table 3). Kaplan–Meier curves demonstrated that the low-dose statin group exhibited a lower rate of cerebrovascular events compared to the no-statin group across various time points (Supplementary Figure 3).

**Table 3:** Multivariate COX regression for cerebrovascular events.

Risk factor	RR (95%CI)	P*
Using low-dose statin	0.392 (0.267–0.574)	**<0.001**
Older	1.026 (1.011–1.042)	**0.001**
Female	1.085 (0.739–1.594)	0.677
Higher NIHSS score at admission	1.076 (1.033–1.121)	**<0.001**
Higher GCS score at admission	0.899 (0.835–0.968)	**0.005**
Higher ADL score at admission	1.011 (0.994–1.028)	0.202
Carotid atherosclerosis	1.756 (1.571–1.986)	**<0.001**
More hematoma at admission	1.031 (0.962–1.105)	0.393
Higher value of PLT at admission	0.996 (0.9994–0.999)	**0.012**
Higher value of albumin at admission	1.029 (1.000–1.058)	**0.047**

P* was calculated by multivariate COX regression. NIHSS, National Institute of Health stroke scale; GCS, Glasgow Coma Scale; ADL, activity of daily living. *P* values highlighted in bold signify statistically significant results.

**Figure 4:**
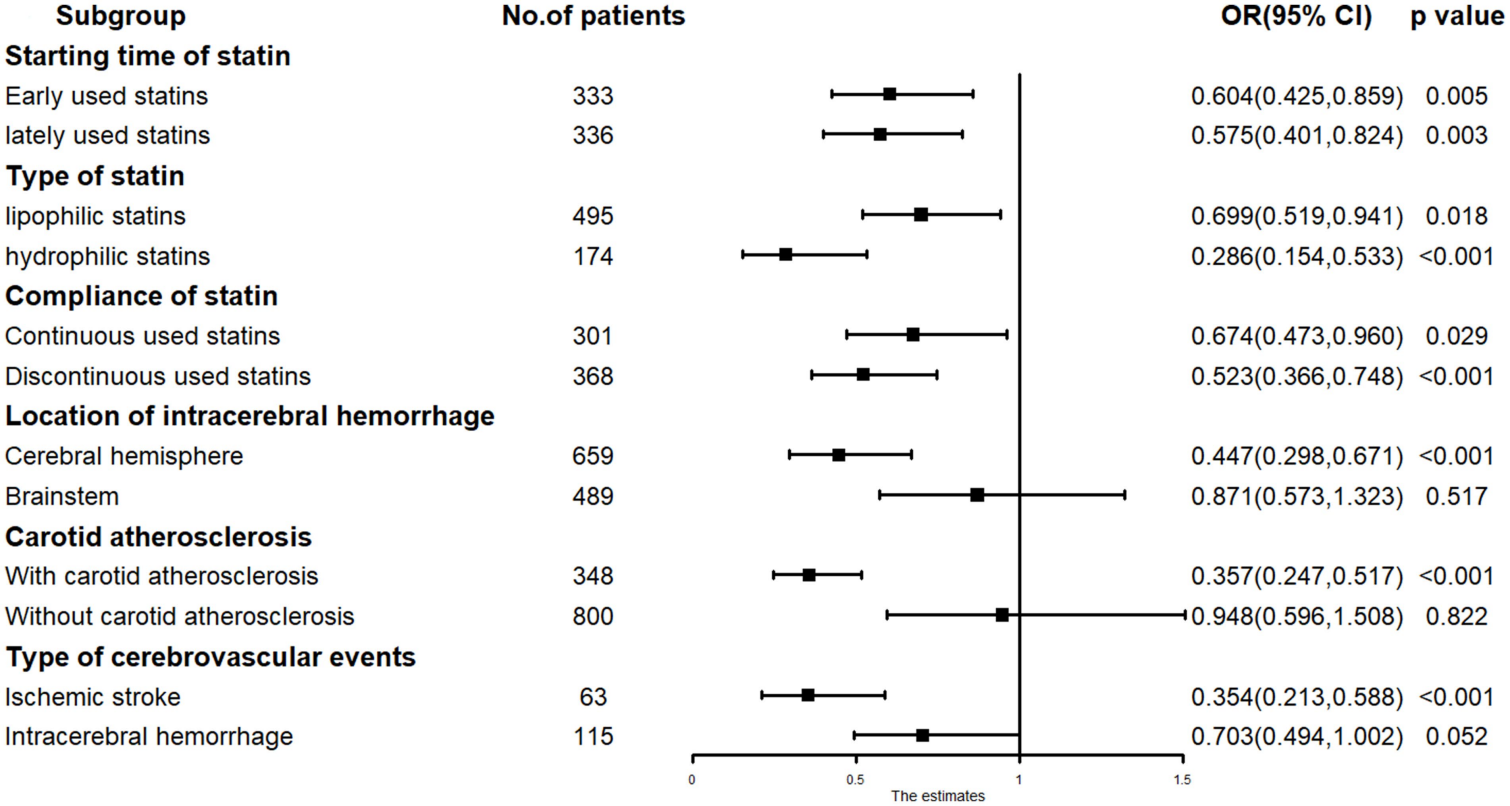
The subgroup analysis results in COX regression: low-dose statin related to cerebrovascular events in carotid atherosclerosis, cerebral hemisphere and ischemic stroke subgroup.

Subgroup analysis revealed that low-dose statin usage (RR = 0.357, *p* < 0.001) was inversely associated with cerebrovascular events in patients with carotid atherosclerosis. Low-dose statin usage (RR = 0.948, *p* = 0.822) showed no association with cerebrovascular events in patients without carotid atherosclerosis. In cases of ICH located in the cerebral hemisphere, low-dose statin usage (RR = 0.447, *p* < 0.001) was inversely associated with cerebrovascular events. In cases of ICH located in the cerebral brainstem, low-dose statin usage (RR = 0.871, *p* = 0.517) showed no significant association with cerebrovascular events. For ischemic stroke occurrence, low-dose statin usage (RR = 0.354, *p* < 0.001) was inversely associated with cerebrovascular events. For ICH recurrence, low-dose statin usage (RR = 0.703, *p* = 0.052) showed no significant association with cerebrovascular events. Significant relationships were observed in other subgroups (Figure 4).

#### Chi-square test analysis for unfavorable functional outcome and hematoma expansion

The low-dose statin group exhibited a lower rate of unfavorable functional outcomes (42.3%) compared to the no-statin group (56.6%) (*p* < 0.001). The low-dose statin group (17.0%) and the no-statin group (16.3%) demonstrated similar rates of hematoma expansion (*p* = 0.735).

#### Logistic regression analysis for unfavorable functional outcome

**Figure 5:**
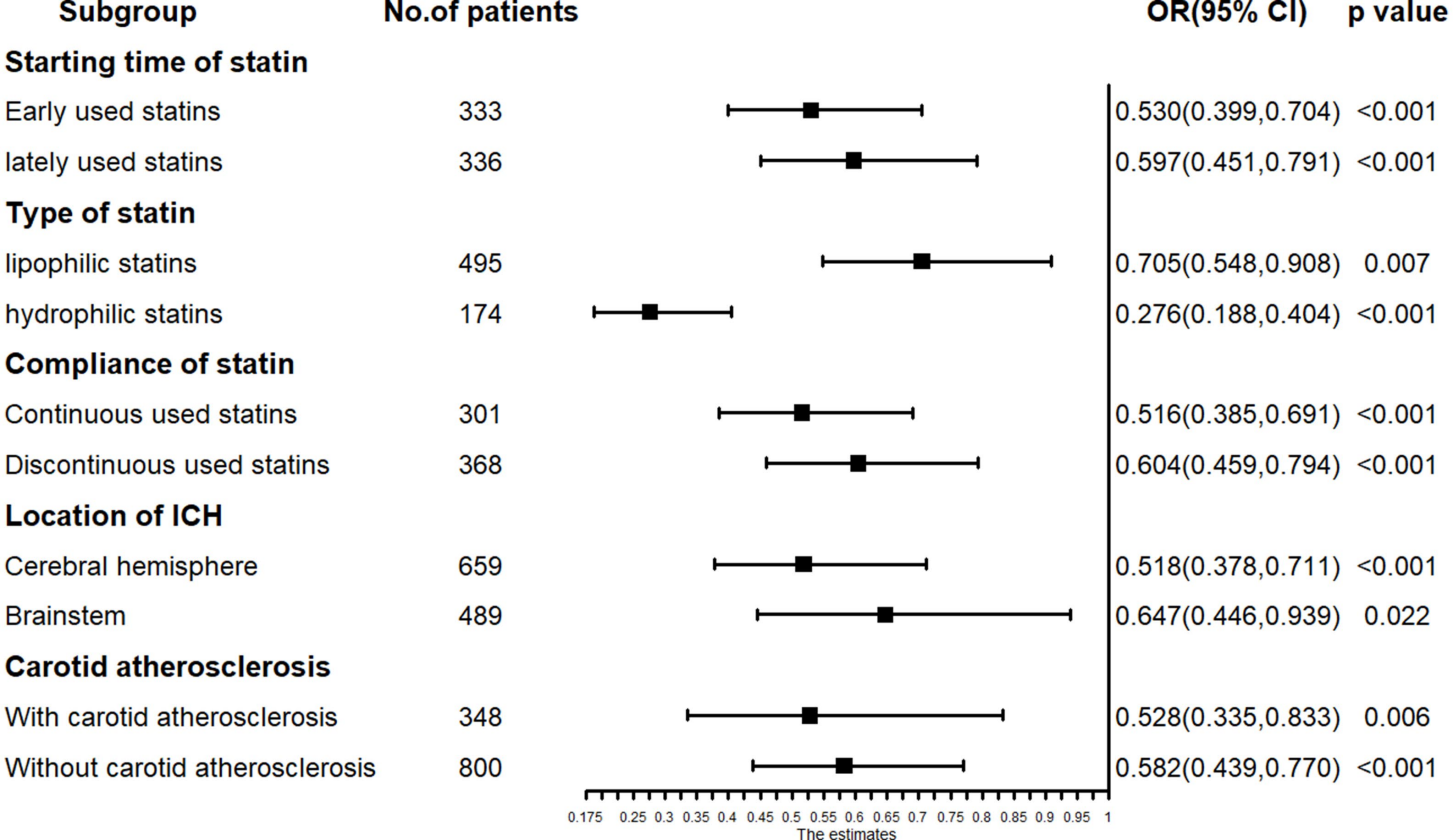
The subgroup analysis results in logistic regression: low-dose statin related to unfavorable function outcome in early-used and lately-used, lipophilic and hydrophilic statins subgroup.

The multivariable logistic regression analysis for unfavorable functional outcome included age, low-dose statin usage, NIHSS score at admission, ADL score at admission, albumin levels at admission, among other factors (Table 4). The results indicated that advanced age (OR = 1.019, *p* = 0.007), higher NIHSS scores (OR = 1.074, *p* = 0.002) at admission, presence of epilepsy (OR = 2.641, *p* = 0.037), and elevated albumin levels (OR = 1.035, *p* = 0.009) at admission were associated with unfavorable functional outcomes. Conversely, low-dose statin usage (OR = 0.594, *p* < 0.001) and higher ADL scores (OR = 0.968, *p* < 0.001) at admission were inversely related to unfavorable functional outcomes (Table 4). Subgroup analysis revealed that early administration of statins (OR = 0.530, *p* < 0.001) and late administration (OR = 0.597, *p* < 0.001) were both inversely associated with unfavorable functional outcomes. Lipophilic statins (OR = 0.705, *p* = 0.007) and hydrophilic statins (OR = 0.276, *p* < 0.001) were inversely associated with unfavorable functional outcomes. Significant relationships were observed in other subgroups (Figure 5).

**Table 4:** Multivariate logistic regression for UFO.

Risk factor	OR (95%CI)	P*
Using low-dose statin	0.594 (0.304–0.861)	**<0.001**
Older	1.019 (1.005–1.032)	**0.007**
Female	1.101 (0.773–1.568)	0.596
Higher NIHSS score at admission	1.074 (1.027–1.123)	**0.002**
Higher GCS score at admission	1.029 (0.935–1.133)	0.561
Higher ADL score at admission	0.968 (0.956–0.980)	**<0.001**
Epilepsy	2.641 (1.059–6.584)	**0.037**
Higher value of glucose at admission	1.028 (0.972–1.087)	0.333
Higher value of albumin at admission	1.035 (1.008–1.062)	**0.009**

P* was calculated by multivariate logistic regression. UFO, unfavorable function outcome; NIHSS, National Institute of Health stroke scale; GCS, Glasgow Coma Scale; ADL, activity of daily living. *P* values highlighted in bold signify statistically significant results.

#### Logistic regression analysis for hematoma expansion

The univariable logistic regression analysis for hematoma expansion indicated that low-dose statin usage (OR = 1.056, *p* = 0.735) was not significantly associated with hematoma expansion. No significant relationships were observed in other subgroups (Supplementary Figure 4). Consequently, multivariable logistic regression analysis was not conducted.

## Discussion

Unsupervised machine learning methods were utilized in the exploratory cohort, revealing that the impact of statins on outcomes was ambiguous. However, analysis of patients on low-dose statins uncovered a clear and significant correlation between statin use and rates of mortality and recurrence. Consequently, a larger prospective validation cohort was utilized to confirm this finding. The validated cohort demonstrated that the low-dose statin group exhibited lower mortality, reduced cerebrovascular event rates, and fewer unfavorable functional outcomes compared to the no-statin group. Both groups exhibited similar rates of hematoma expansion. Univariable COX and logistic regression analyses indicated that low-dose statin use was associated with lower mortality, reduced cerebrovascular events, and a decreased rate of unfavorable functional outcomes. In the mortality analysis, no association was observed between statins and outcomes in subgroups with later statin use, those using lipophilic statins, and those with ICH located in the cerebral brainstem. Conversely, in other subgroups, statins were associated with outcomes. In the cerebrovascular events analysis, no association was found between statins and outcomes in subgroups of patients without carotid atherosclerosis, those with ICH located in the cerebral brainstem, and those with ICH recurrence. Conversely, statins were associated with outcomes in other subgroups. Logistic regression analysis indicated that low-dose statin use was not associated with hematoma expansion.

### Unsupervised machine learning methods were employed to explore unknown factors potentially impacting outcomes

Unsupervised machine learning techniques were applied to analyze unlabeled data, aiming to identify unknown factors potentially influencing outcomes. In prior research, this approach was utilized to investigate strategies for mitigating side effects of statins and antiplatelet drugs in patients with ischemic stroke (10, 11). A recent study utilized this method to examine factors affecting the prognosis of ischemic stroke patients undergoing transcranial magnetic stimulation therapy (12). While traditional analysis of the exploratory cohort suggested statins influenced cerebral hemorrhage prognosis without significant effects, unsupervised learning revealed ambiguous correlations between statins and outcomes, indicating that statin type might play a role in the observed results. Further analysis of patients on low-dose statins uncovered a statistically significant association between statin use and patient prognosis. Unsupervised scatter plots and heat maps illustrated a clear and significant relationship between low-dose statins and adverse outcomes, including mortality and recurrence, in patients with cerebral hemorrhage. This correlation was subsequently validated in the prospective cohort.

### Mortality and unfavorable function outcome

Some studies have suggested that normal-dose statins are associated with lower all-cause mortality and better functional outcomes in ICH patients (4, 7, 13). Other studies have shown that statin use is not associated with mortality or functional outcomes (2, 14). Our results indicate that low-dose statins are also effective in reducing mortality and improving functional outcomes in ICH patients. Differences in the number of patients on low-dose statins and the type of cohorts might explain the varying results. One study suggested that early statin use could worsen the condition of ICH patients (4). We found that early statin use decreases mortality in ICH patients. ICH patients using statins early could benefit from lower mortality. For ICH located in the cerebral brainstem subgroup, low-dose statin use was not associated with mortality or unfavorable functional outcomes. The more severe conditions and higher mortality in these patients could explain these results. Low-dose statin use was not associated with unfavorable functional outcomes in patients without carotid atherosclerosis. This finding suggests that the mechanism by which statins improve functional outcomes may be anti-atherosclerotic. Patients could benefit from statins through multiple mechanisms over an extended period. Therefore, statins could affect the 3-year mortality of ICH patients with or without carotid atherosclerosis.

### Cerebrovascular events

Low-dose statin use was associated with reduced cerebrovascular events (4, 15). Our findings demonstrated a significant association with ischemic stroke occurrence but not with ICH recurrence. These results are consistent with previous studies. Two studies also found no association between statin use and ICH recurrence (16, 17). However, two other studies indicated that statin use decreased the rate of ICH recurrence (8, 15). Differences in cohort characteristics and statin dosage may partly explain these discrepancies.

A higher albumin value was associated with cerebrovascular events, which is a contradictory finding. Additionally, a higher albumin value was also linked to unfavorable functional outcomes. Patients with lower albumin levels might have been in more severe condition, thereby having a reduced chance of survival regarding outcome events. This may partially explain the findings.

### Hematoma expansion

One study demonstrated an association between early use of normal-dose statins and hematoma expansion (18). Another study indicated that lower LDL-C levels were associated with hematoma expansion (5). However, our findings revealed no association between low-dose statin use and hematoma expansion (19). Differences in statin dosage and LDL-C levels may explain the discrepancies between our study and others (18–20). The association was not significant in both early-use and later-use statin subgroups. Across different types of statin subgroups, no significant associations were observed. The association was also not significant across various ICH location subgroups. Therefore, for ICH patients, low-dose statins may be safer than normal-dose statins, particularly when used early.

### Limitation

This study was a cohort design, which did not allow for randomization of patient groups. Nevertheless, the findings from this cohort study are more reflective of real-world patient conditions. Additionally, multivariate and subgroup analyses were employed to validate the robustness of our findings.

## Conclusion

Unsupervised machine learning techniques identified a correlation between low-dose statin administration and the prognosis of patients with cerebral hemorrhage. The validated cohort revealed that for patients with ICH in the cerebral hemisphere, early administration of low-dose hydrophilic statins was associated with reduced mortality. In patients with ICH in the cerebral hemisphere, low-dose statins were linked to a decreased incidence of ischemic stroke. Low-dose statins were also associated with a reduced rate of unfavorable functional outcomes in ICH patients. Among ICH patients, low-dose statins showed no association with hematoma expansion. This research employed unsupervised machine learning approaches to analyze risk factors in complex clinical conditions, such as cerebral hemorrhage, thereby facilitating the development of more precise preventive measures and personalized therapeutic strategies.

## Data Availability

The raw data supporting the conclusions of this article will be made available by the authors, without undue reservation.
