# Supplementary material for: Unsupervised machine learning revealed a correlation between low-dose statins and favorable outcomes in ICH patients
Source: Front Neurol. 2025 Jun 24;16:1573036. doi: 10.3389/fneur.2025.1573036 (PMC12241808; doi:10.3389/fneur.2025.1573036)
Supplement: Supplementary file 1 [file Table_1.docx]

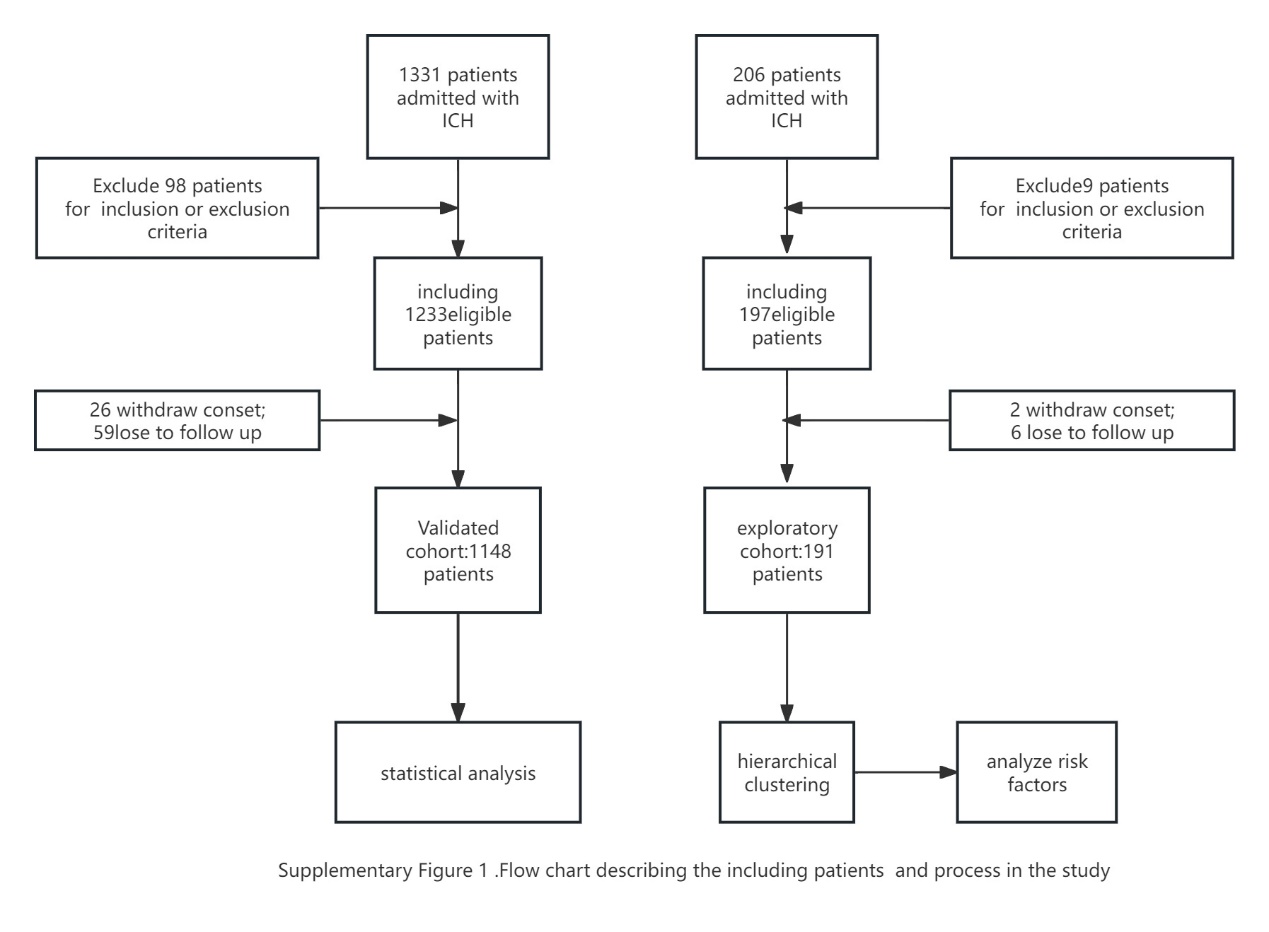


Supplementary-figure 01: Flowchart describing including patients in two cohort


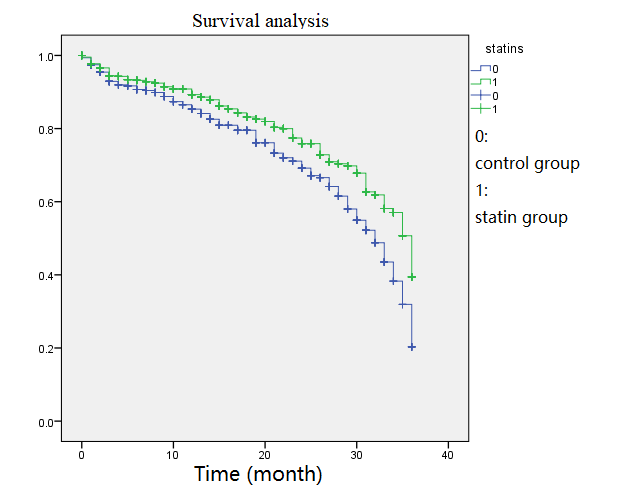


Supplementary-figure 02: the K-M curves: difference survival rate within 36 months between two groups


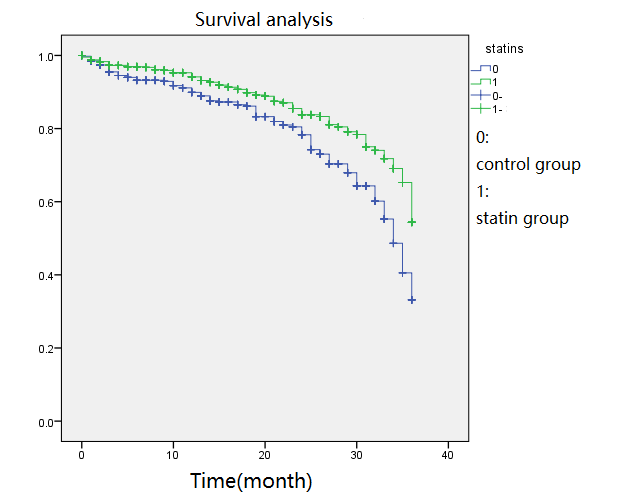


Supplementary-figure 03: the K-M curves: difference cerebrovascular events within 36 months between two groups


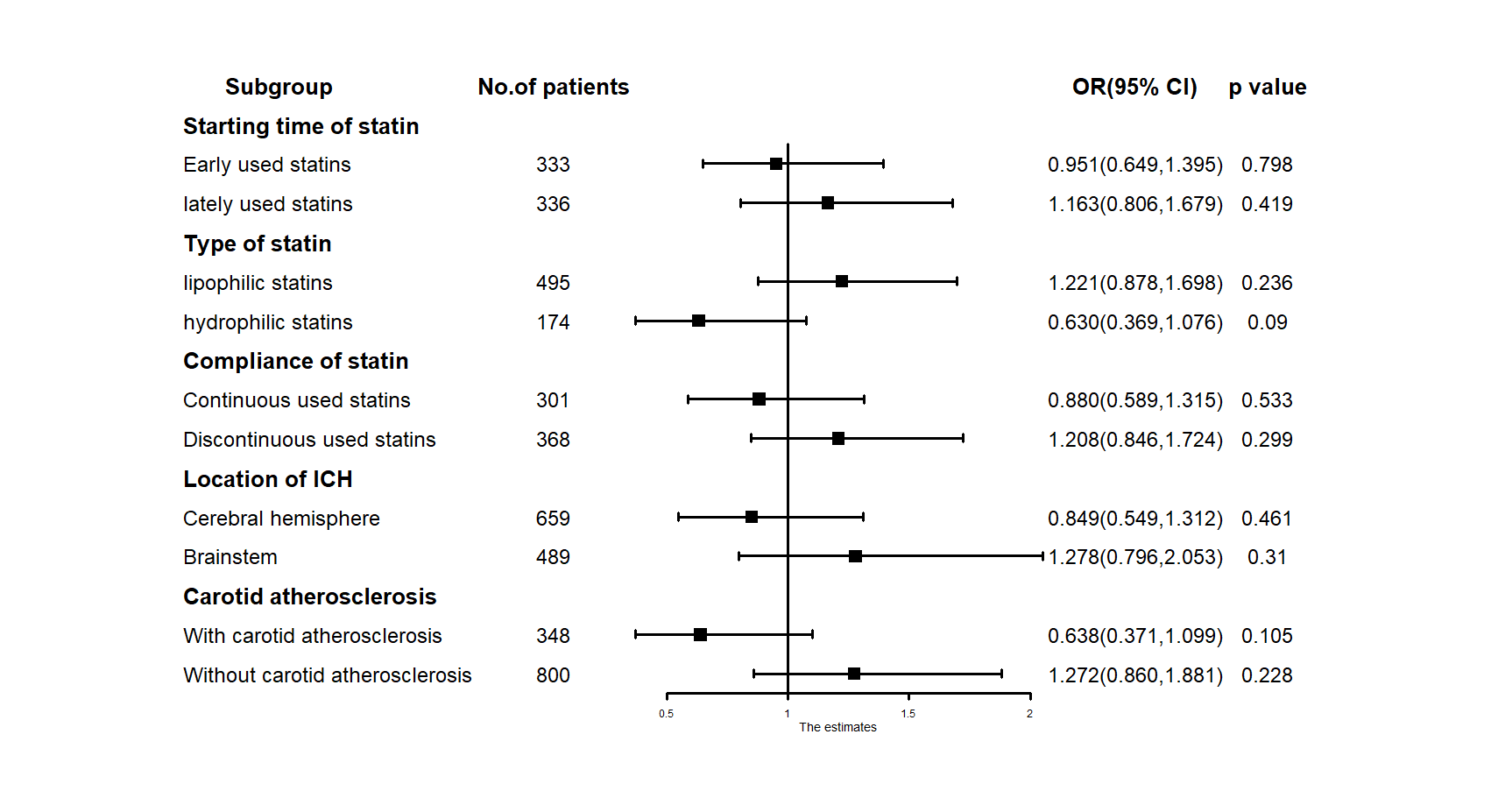


Supplementary-figure 04: The subgroup analysis results in logistic regression: relationship (OR) between low dose statin and hematoma expansion
