# Supplementary material for: Unsupervised machine learning revealed a correlation between low-dose statins and favorable outcomes in ICH patients
Source: Front Neurol. 2025 Jun 24;16:1573036. doi: 10.3389/fneur.2025.1573036 (PMC12241808; doi:10.3389/fneur.2025.1573036)
Supplement: Supplementary file 2 [file Table_2.docx]

# 1、data

# In[1]:

import numpy as np

import pandas as pd

# In[2]:

data = pd.read_excel("e:/ICHstatin1dead.xlsx",index_col=0)

# In[3]:

data_ = data.copy()

data_

# In[4]:

data_.info()

# # 2、set

# In[5]:

features = data_.drop(columns=['dead'])

features

# In[6]:

target = data_.dead.replace(to_replace = [0,1], value = [0,1])

target

# In[7]:

from sklearn.preprocessing import StandardScaler

# In[8]:

transfer = StandardScaler()

# In[9]:

features_ = transfer.fit_transform(features)

# In[10]:

features_

# In[11]:

#features_ = features_[:,:9]

# # 3、K-means

# In[12]:

from sklearn.decomposition import PCA

# In[13]:

pca=PCA()

pca_2 = PCA(n_components=2)

pca_2

pca.components_

# In[14]:

features_2 = pca_2.fit_transform(features)

# In[15]:

features_2.shape

# In[16]:

x=features_2[:,0]

y=features_2[:,1]

x

y

# In[17]:

import matplotlib.pyplot as plt

plt.scatter(x,y)

plt.plot(pca_2.components_,marker='o')

# In[18]:

from sklearn.cluster import KMeans

# In[19]:

kmeans = KMeans(n_clusters=2)

# In[20]:

y_kmeans = kmeans.fit_predict(features_)

# In[21]:

y_kmeans

# In[22]:

plt.scatter(x[y_kmeans==0],y[y_kmeans==0])

plt.scatter(x[y_kmeans==1],y[y_kmeans==1])

plt.scatter(x[y_kmeans==2],y[y_kmeans==2])

plt.show()

# In[23]:

clusters = np.arange(2,11)

clusters

# In[24]:

inertia=[]

for i in clusters:

kmeans = KMeans(n_clusters=i)

kmeans.fit(features_)

inertia.append(kmeans.inertia_)

# In[25]:

inertia

# In[26]:

plt.plot(clusters,inertia,"bo-")

# In[27]:

from sklearn.metrics import silhouette_score

# In[28]:

sil_score=[]

for i in clusters:

kmeans = KMeans(n_clusters=i)

kmeans.fit(features_)

sil_score.append(silhouette_score(features_,kmeans.labels_))

# In[29]:

plt.plot(clusters,sil_score,"bo-")

# # 4、DBSCN

# In[30]:

from sklearn.cluster import DBSCAN

# In[31]:

dbscan = DBSCAN()

# In[32]:

y_db = dbscan.fit_predict(features_)

# In[33]:

y_db

# In[34]:

eps_ = np.linspace(0.5,2,100)

# In[35]:

sil_score=[]

for i in eps_:

dbscan = DBSCAN(eps=i)

dbscan.fit(features_)

sil_score.append(silhouette_score(features_,dbscan.labels_))

# In[36]:

plt.plot(eps_,sil_score)

# In[37]:

index_ = np.argmax(sil_score[:80])

best_eps = eps_[index_]

dbscan_ = DBSCAN(eps=best_eps)

# In[38]:

y_db_ = dbscan_.fit_predict(features_)

# In[39]:

y_db_

# In[40]:

plt.scatter(x[y_db_==0],y[y_db_==0])

plt.scatter(x[y_db_==1],y[y_db_==1])

plt.scatter(x[y_db_==-1],y[y_db_==-1])

plt.show()

# # 5、Gaussian Mixture

# In[41]:

from sklearn.mixture import GaussianMixture

# In[42]:

gm = GaussianMixture(n_components=2, n_init=10)

# In[43]:

y_gm = gm.fit_predict(features_)

# In[44]:

plt.scatter(x[y_gm==0],y[y_gm==0])

plt.scatter(x[y_gm==1],y[y_gm==1])

plt.scatter(x[y_gm==2],y[y_gm==2])

plt.show()

# In[45]:

AIC=[]

BIC=[]

for i in clusters:

gm = GaussianMixture(n_components=i,n_init=10)

gm.fit(features_)

AIC.append(gm.aic(features_))

BIC.append(gm.bic(features_))

# In[46]:

plt.plot(clusters,AIC,'b')

plt.plot(clusters,BIC,'r')

# # 6、hierarchical clustering

# In[47]:

import seaborn as sns

# In[48]:

sns.set(font_scale=1.8)

plt.figure(figsize=(90, 90))

plt.subplots(figsize=(90, 90))

sns.clustermap(features_,method='ward')

# In[49]:

sns.clustermap(features,method='ward',standard_scale=True)

# In[50]:

from sklearn.preprocessing import MinMaxScaler

mm_transfer = MinMaxScaler()

features_minmax = mm_transfer.fit_transform(features)

# In[51]:

sns.clustermap(features_minmax,method='ward')

from sklearn.cluster import AgglomerativeClustering

from scipy.cluster.hierarchy import fcluster

import numpy as np

cluster = AgglomerativeClustering(n_clusters=2)

labels = cluster.fit_predict(features_)

n_clusters = 2 #

cluster_method = 'distance' # 或者 'single'

max_distance = 0.3 # 当cluster_method为'distance'

#

cluster_assignment = fcluster(features_, t=max_distance, criterion=cluster_method)

#

print(cluster_assignment)

# In[52]:

from sklearn.cluster import AgglomerativeClustering

# In[53]:

aggcluster = AgglomerativeClustering(n_clusters=2,linkage='ward')

# In[54]:

y_agg = aggcluster.fit_predict(features_minmax)

# In[55]:

y_agg
